# Risk of Insulin Resistance in 44,939 Spanish Healthcare Workers: Association with Sociodemographic Variables and Healthy Habits

**DOI:** 10.3390/diseases13020033

**Published:** 2025-01-27

**Authors:** Pedro Javier Tárraga Marcos, Ángel Arturo López-González, Emilio Martínez-Almoyna Rifá, Hernán Paublini Oliveira, Cristina Martorell Sánchez, Pedro Juan Tárraga López, José Ignacio Ramírez-Manent

**Affiliations:** 1 Sant Joan University Hospital, 03550 Alicante, Spain; pedrojav2003@gmail.com; 2 ADEMA-Health Group of University, Institute of Health Sciences (IUNICS) of Balearic Islands, 07009 Palma, Spain; emilio@udemax.com (E.M.-A.R.); h.paublini@eue.edu.es (H.P.O.); c.martorell@eua.edu.es (C.M.S.); joseignacio.ramirez@ibsalut.es (J.I.R.-M.); 3 Faculty of Odontology, University School ADEMA-UIB, 07009 Palma, Spain; 4 Health Service of the Balearic Islands, 07120 Palma, Spain; 5 Faculty of Medicine, Castilla la Mancha University, 02008 Albacete, Spain; pjtarraga@sescam.jccm.es; 6 Faculty of Medicine, Balearic Islands University, 07120 Palma, Spain

**Keywords:** insulin resistance, TyG index, Mediterranean diet, healthcare worker, physical activity, tobacco consumption

## Abstract

**Introduction:** Insulin resistance (IR) is a highly prevalent pathophysiological entity implicated in the development of a wide variety of metabolic, cardiovascular, and endocrine disorders. The aim of this study is to assess the association between sociodemographic variables and healthy habits with IR risk scales. **Methodology:** This dual study, incorporating both longitudinal-retrospective and cross-sectional designs, analyzed healthcare workers across four professional categories (physicians, nurses, healthcare technicians, and auxiliary personnel). It examined the association of age, sex, professional category, smoking status, physical activity, and adherence to the Mediterranean diet with elevated scores on insulin resistance risk scales. **Results:** All the variables analyzed were associated with the presence of elevated values of the IR scales, with age, sex, and physical activity showing the strongest association (reflected in the odds ratio values). **Conclusions:** The profile of an individual with a higher risk of presenting elevated values of the IR risk scales would be an elderly male auxiliary health worker who is a smoker and is physically inactive, with a low adherence to the Mediterranean diet.

## 1. Introduction

Insulin resistance (IR) is a fundamental pathophysiological alteration implicated in the development of a wide range of metabolic, cardiovascular, and endocrine disorders. This condition, characterized by a decreased ability of peripheral tissues to respond adequately to insulin, plays a crucial role in the onset of conditions such as metabolic syndrome [1], type 2 diabetes mellitus (T2DM) [2], and other related conditions, including hypertension [3], dyslipidemia [4], and cardiovascular disease (CVD) [5]. Given the global increase in obesity and associated metabolic diseases, and their relationship with the pathogenesis of type 2 diabetes mellitus T2DM, understanding the pathophysiological basis of insulin resistance (IR), its clinical implications, diagnostic methods, and epidemiological distribution is essential for designing effective strategies for prevention, early detection, and treatment [6].

IR arises from an imbalance in the interaction between insulin signaling and the ability of target cells, particularly in skeletal muscle, adipose tissue, and the liver, to respond to this hormone [7]. Under normal conditions, insulin facilitates glucose uptake by mobilizing GLUT4 transporters to the cell membrane [8], a process particularly significant in skeletal muscle, as this tissue accounts for most of the glucose clearance following food intake [9]. In individuals with IR, this mechanism is impaired due to dysfunctions in the intracellular signaling pathways, associated with factors such as low-grade inflammation [10], oxidative stress [11], intramyocellular lipid accumulation [12], and alterations in the gut microbiota [13].

A central element in individuals with IR is the elevated presence of free fatty acids (FFAs) [14] and lipid intermediates, such as ceramides [15] and diacylglycerols [16]. These components directly interfere with insulin signaling pathways at the receptor level and their intracellular effectors, such as IRS-1 (insulin receptor substrate-1) [17] and PI3K (phosphatidylinositol-3-kinase) [18]. Additionally, imbalances in adipokines, such as adiponectin and leptin, exacerbate this dysfunction, particularly in obesity contexts [19].

At the hepatic level, IR results in increased gluconeogenesis and a reduced capacity to inhibit hepatic glucose production, contributing to fasting hyperglycemia [20]. In adipose tissue, impaired insulin signaling leads to uncontrolled lipolysis, releasing FFAs into the bloodstream and worsening IR in other tissues [21]. Finally, in skeletal muscle, reduced glucose uptake is a key factor in the development of glucose intolerance [22].

Diagnosing IR presents challenges due to the absence of a direct, universally accepted marker. Although the hyperinsulinemic–euglycemic clamp is considered the gold standard for measuring insulin sensitivity, its technical complexity and cost restrict its use to research settings primarily [23]. Consequently, clinical practice relies on indirect methods using biochemical indicators and mathematical models.

Widely used methods include the HOMA-IR (Homeostatic Model Assessment for Insulin Resistance), which is calculated from fasting glucose and insulin levels [24]. In recent years, more accessible and robust indices have emerged, such as the Triglyceride–Glucose (TyG) index, SPISE-IR (Single-Point Insulin Sensitivity Estimator), and METS-IR (Metabolic Score for Insulin Resistance):

- TyG index: this index has demonstrated a strong correlation with the hyperinsulinemic clamp and is validated as a reliable predictor of diabetes, metabolic syndrome, and cardiovascular risk [25].
- SPISE-IR: this index is designed to estimate insulin sensitivity in non-diabetic populations and is highly useful for detecting IR in individuals with obesity [26].
- METS-IR: this score reflects the overall metabolic status and is helpful for identifying IR and stratifying the risk of metabolic complications across diverse populations [27].

These indices have proven to be practical and reliable tools, particularly in resource-limited settings. However, their interpretation must be complemented with a comprehensive clinical evaluation that considers family history, lifestyle habits, and the presence of comorbidities.

The prevalence of IR varies widely among populations depending on diagnostic methods and criteria. Globally, a significant proportion of the population exhibits some degree of IR, with higher rates among individuals with obesity [28], metabolic syndrome [29], or a family history of diabetes [30].

Obesity is associated with various risk factors, including sociodemographic characteristics and lifestyle habits. Among these, age stands out, as several studies have reported an increase in obesity prevalence with aging [31]. Sex also plays a role, with individual differences influenced by hormones [32], such that men exhibit greater visceral obesity at younger ages [33], whereas women experience a significant increase in abdominal fat after menopause [34]. Socioeconomic status is another key factor, with numerous studies finding a higher prevalence of obesity in lower socioeconomic classes [35].

Among lifestyle habits, recent studies have linked smoking to obesity, elevated triglyceride levels, and reduced HDL cholesterol levels [36]. Physical exercise has proven effective in the prevention and management of at least 23 diseases. However, the adherence to regular physical activity remains low across populations, with studies highlighting this challenge among healthcare workers particularly, especially those working in shifts [37]. The last variable evaluated in our study is the adherence to the Mediterranean diet. Excess calorie intake and diets high in fats and sugars are strongly associated with obesity. The Mediterranean diet, characterized by a high intake of fruits, vegetables, legumes, nuts, fish, and olive oil, has been shown to reduce systemic inflammation, decrease abdominal obesity, and prevent other metabolic disorders [38]. These factors have been shown to be associated with obesity in the general population, which, as discussed, is closely linked to insulin resistance (IR).

Healthcare workers face additional occupational stressors that significantly impact their risk behaviors and overall health. Long working hours limit their time for rest and recovery, while the constant exposure to suffering and life-or-death situations generates continuous emotional strain. Furthermore, the pressure to meet public expectations, professional conflicts with patients and colleagues, and the constant need to stay updated with technological and scientific advancements contribute to elevated workplace stress levels.

Other factors, such as staff shortages, lead to physical and mental overexertion, while shift work disrupts natural sleep and rest cycles, resulting in metabolic imbalances. These conditions often promote unhealthy habits, such as poor diet, physical inactivity, or the increased use of harmful substances like tobacco or alcohol. These behaviors not only increase the risk of developing obesity and insulin resistance but also significantly elevate cardiometabolic risk [39]. Therefore, recognizing and addressing these stressors is essential to implementing strategies that promote the physical and mental well-being of healthcare workers, ensuring their ability to perform a vital role in society.

This study aims to evaluate how sociodemographic variables such as age, sex, and socioeconomic status, as well as healthy habits like smoking, physical activity, and adherence to the Mediterranean diet, are associated with scales assessing the risk of insulin resistance, including the TyG index, SPISE-IR, and METS-IR.

## 2. Methods

### 2.1. Study Design and Sample

This study utilized a mixed-methods approach, combining a retrospective longitudinal study and a cross-sectional descriptive study. A total of 44,939 healthcare workers from various regions of Spain participated. The sample comprised 14,305 men (31.8%) and 30,634 women (68.2%). The participants were selected from individuals undergoing mandatory annual medical check-ups provided by their employers during the study period. The longitudinal study covered the time frame from 2010 to 2019.

#### 2.1.1. Inclusion Criteria

Aged between 18 and 69 years.
Employed by one of the participating companies.
Provided informed consent to participate in the study.
Authorized the use of their data for epidemiological purposes.

#### 2.1.2. Exclusion Criteria

Age under 18 or over 69 years.
No employment contract with a participating company.
Did not provide informed consent to participate in the study.
Did not authorize the use of their data for epidemiological purposes.

**Figure 1:**
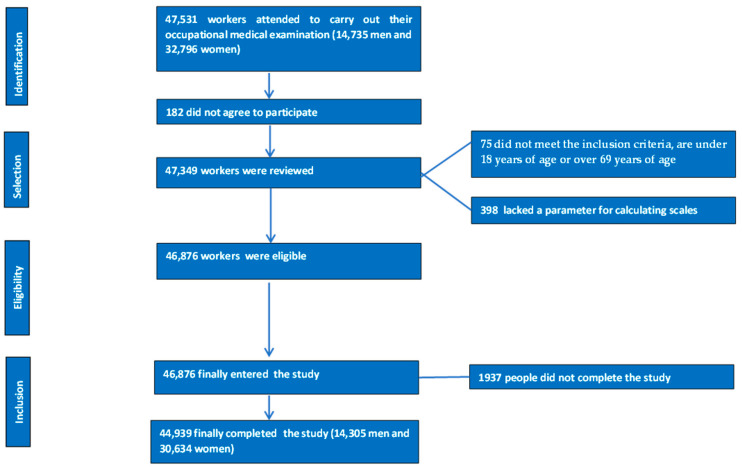
The flowchart detailing the selection and inclusion process for study participants.

The flowchart of study participants is presented in Figure 1.

### 2.2. Data Collection Procedures

Data collection was conducted by occupational health teams from the collaborating companies using the following methodologies:

- Medical History: sociodemographic information (e.g., age, gender, occupation) and health-related data, such as smoking status, physical activity levels, adherence to the Mediterranean diet, and stress levels, were gathered.
- Physical and Clinical Measurements: parameters including height, weight, waist circumference, hip circumference, and systolic and diastolic blood pressure were recorded.
- Laboratory Tests: biochemical variables, such as lipid profiles, liver function markers, and fasting blood glucose levels, were analyzed.

To minimize bias, all measurements followed standardized protocols:

- Height and Weight: measured using a SECA 700 scale and a SECA 220 stadiometer (SECA, Chino, CA, USA), with participants dressed only in underwear.
- Circumferences: Waist circumference was measured using a SECA measuring tape, positioned midway between the lowest rib and the iliac crest. Hip circumference was measured at the widest point of the buttocks, with participants standing upright and relaxed.
- Blood Pressure: Taken with an OMRON-M3 sphygmomanometer (OM RON, Osaka, Japan) after 10 min of rest in a seated position. Participants were instructed to abstain from food, beverages, and tobacco for at least one hour prior. Three measurements were taken at one-minute intervals, and the average was calculated.

Blood samples were collected via venipuncture after a 12 h fast, refrigerated, and processed in reference laboratories within 72 h. The analyses included the following:

- Triglycerides, total cholesterol, and glucose: measured using enzymatic methods.
- HDL cholesterol: measured using a precipitation method.
- LDL cholesterol: calculated using the Friedewald formula when triglycerides were below 400 mg/dL.

The following insulin resistance risk scales were applied:

- TyG index [40]: calculated as TyG = LN (triglycerides × glycemia/2), with values of 8.5 or higher considered as high risk.
- Single-Point Insulin Sensitivity Estimator (SPISE): Calculated as SPISE = (600 × HDL^0.185)/(triglycerides^0.2 × BMI^1.338). SPISE-IR = 10/SPISE high-risk values are defined as 1.51 or above [41].
- Metabolic Score for Insulin Resistance (METS-IR) [42]: Calculated as METS-IR = LN(2 × glucose) + (triglycerides × BMI)/LN(HDL-c). High-risk values are defined as 50 or above.

### 2.3. Operational Definitions

- Professional Categories: healthcare workers were classified into four groups: physicians, nurses, health technicians (laboratory, pathology, and radiology), and nursing assistants or orderlies.
- Smoking: defined as consuming at least one cigarette per day within the past 30 days or having quit smoking within the past year.
- Mediterranean Diet Adherence: assessed using the PREDIMED questionnaire, with high adherence classified as a score of 9 or higher [43].
- Physical Activity: measured using the International Physical Activity Questionnaire (IPAQ), evaluating the frequency, duration, and intensity of physical activity [44].

### 2.4. Statistical Analysis

Descriptive analyses of categorical variables were conducted using frequencies and distributions. The Kolmogorov–Smirnov test was applied to assess the normality of quantitative variables, followed by calculations of means and standard deviations. For the bivariate analysis, Student’s *t*-test was used to compare means, and the chi-square test was employed to assess proportions.

The variables associated with metabolic syndrome (MS) and high triglyceride-waist phenotype (HTW) were analyzed using a binary logistic regression model. The goodness-of-fit of the model was evaluated with the Hosmer–Lemeshow test. A stratified analysis was performed to identify potential confounding factors; however, no significant confounders were detected. Pearson’s and Cohen’s kappa coefficients were used to assess the correlation and agreement between the IR risk scales. Statistical analyses were performed using SPSS software version 29.0, with a significance threshold of 0.05.

### 2.5. Ethical Considerations

This study complied with the ethical principles outlined in the Declaration of Helsinki and was approved by the Ethics and Research Committee of the Balearic Islands (CEI-IB) under protocol code IB 4383/20. All participants provided signed informed consent, and their data were anonymized in accordance with Spain’s Organic Law 3/2018 on Data Protection.

## 3. Results

The anthropometric, clinical, analytical, sociodemographic, and lifestyle data of the 44,939 healthcare workers included in the study are presented in Table 1. The participants’ mean age was slightly over 41 years, with the majority falling between 30 and 49 years of age. Across all variables, lower values were observed in the female group.

Adherence to the Mediterranean diet was reported by 45.8% of men and 37.9% of women, while 47.5% of men and 38.9% of women engaged in regular physical activity. Smoking prevalence was slightly higher among men, with 16.1% of male participants and 15% of female participants reporting smoking.

Table 2 and Table 3 present the mean values and prevalence of elevated scores for the three IR risk scales according to various sociodemographic variables and healthy habits. The data reveal that both mean values and the prevalence of high scores increase progressively with age. Similarly, this upward trend is observed as socioeconomic status decreases, among smokers, individuals who do not engage in regular physical activity, and those with a low adherence to the Mediterranean diet. Both mean values and the prevalence of high scores are lower in women. In all cases, the observed differences demonstrate high statistical significance (*p* < 0.001).

Table 4 presents the results of the binary logistic regression analysis. All the sociodemographic variables and healthy habits analyzed are associated with the presence of elevated scores on the three IR risk scales. Among these, the variables showing the strongest associations, as indicated by the highest odds ratio values, are age, sex, and physical activity.

Table 5 presents the results of Pearson’s correlation coefficients and Cohen’s kappa concordance values for the different IR scales. Moderate values are observed for TyG, compared to SPISE-IR and METS-IR, in both coefficients, while high values are observed for METS-IR and SPISE-IR, also in both coefficients.

Table 6 presents the results of the retrospective longitudinal study conducted between 2010 and 2019. It shows that the differences in the prevalence of the three IR scales between the two periods increase with age. This same upward trend is also observed as socioeconomic status decreases, among smokers, sedentary individuals, and those who do not regularly follow a Mediterranean diet.

## 4. Discussion

In our study, all analyzed variables, especially age, sex, and physical activity, are associated with elevated values in the insulin resistance (IR) risk scales examined.

Age, as previously discussed, is a factor associated with a higher risk of IR in our study. As people age, insulin sensitivity decreases, partly due to the accumulation of visceral fat [45], loss of muscle mass [46], and mitochondrial dysfunction [47]. Several studies confirm our results, having documented a higher prevalence of IR in older individuals. According to a study by Koh-Banerjee, the prevalence of IR increases significantly after the age of 40, driven by the changes in fat distribution and decreased physical activity [48]. This trend has been observed across various worker cohorts, including healthcare professionals, where older age groups exhibit higher IR indices compared to their younger counterparts [49].

Sex also appears to play an important role in the predisposition to IR, aligning with our results. These findings are consistent with other studies reporting that men have higher IR levels than women, although this pattern is influenced by hormonal factors, fat distribution, and physical activity. A study of the working population in Taiwan found that men are at a greater risk of developing IR due to a higher amount of visceral fat and lower insulin sensitivity [50]. However, in postmenopausal women, hormonal changes can exacerbate IR, putting them at greater risk as they age [51]. Among healthcare workers, women generally exhibit lower smoking rates and higher engagement in exercise, which may contribute to the lower prevalence of IR in this group compared to other occupational sectors [52].

Socioeconomic status (SES) also appears to be a significant determinant of metabolic health, based on our findings. This aligns with studies demonstrating an inverse relationship between SES and IR prevalence. Individuals with a lower SES often have limited access to healthcare, healthy food, and recreational activities, as highlighted in a Finnish study [53]. The research by Sánchez-Rodríguez found that workers in low-SES roles, including some healthcare workers in lower-ranking positions, face a higher risk of IR, associated with their poor diet, higher job stress, and reduced time for physical activity [54]. Another study also highlighted the association between SES and IR, assessed using the TyG index [55].

Smoking is a risk factor that is negatively associated with IR in our study. Chemicals in tobacco promote systemic inflammation, disrupt endothelial function, and impair the body’s ability to manage glucose [56]. However, an Indian study did not observe this association [57]. Among healthcare workers, smoking remains prevalent despite awareness campaigns. A study on Spanish healthcare professionals showed a strong relationship between smoking and IR, with smokers more likely to develop type 2 diabetes [58].

Physical activity emerged as a factor associated with a lower risk of IR, which is consistent with the well-established role of exercise in improving insulin sensitivity. Numerous studies support our findings that regular physical activity, particularly aerobic and resistance exercise, enhances glucose uptake by muscles and reduces visceral fat accumulation [59,60]. A study on Spanish healthcare workers found that physically active individuals had lower IR levels, regardless of age or gender [53]. Among healthcare professionals, physical exercise is often used as a strategy to counteract the effects of occupational stress, sedentary lifestyles, and poor dietary habits. However, long work hours and high job demands can hinder the adoption of healthy habits. A study revealed that despite knowledge about the benefits of exercise, a significant proportion of healthcare workers fail to meet exercise recommendations, leading to a higher prevalence of IR in this group [61].

A high adherence to the Mediterranean diet was associated, in our study, with a lower prevalence of IR. The Mediterranean diet has been widely recognized as an effective dietary approach to improve insulin sensitivity. Rich in antioxidants, healthy fats (such as olive oil), and fiber, this diet has protective effects on glucose metabolism and reduces chronic inflammation—an important factor in IR [62]. A systematic review and meta-analysis found that the adherence to the Mediterranean diet is inversely associated with IR, even in individuals with a genetic predisposition to type 2 diabetes [63]. For healthcare workers, the Mediterranean diet may offset the negative effects of occupational stress and irregular meal patterns. However, many healthcare professionals, particularly those working long or night shifts, tend to have less healthy diets, increasing their risk of IR [64].

The use of indices, such as TyG, SPISE-IR, and METS-IR, has gained popularity for indirectly assessing IR. The TyG index is a reliable marker for estimating IR, particularly in individuals with an altered metabolic profile. A study validated the use of the TyG index for assessing IR, showing that higher triglyceride–glucose ratios are closely linked to an increased risk of type 2 diabetes and cardiovascular disease [65]. Among workers, the TyG index has been successfully used to evaluate IR risk [66,67].

The SPISE-IR index, which assesses sarcopenia and functional capacity in older adults, has also been linked to IR, as a lower muscle mass and impaired physical function are contributing factors to IR [68]. Lastly, the METS-IR index, which incorporates factors such as hypertension and lipid levels, has been used to evaluate the metabolic risk related to IR in various cohorts, including healthcare workers [69].

In contemporary society, healthcare demands and needs are exponentially increasing, driven by factors such as population aging, the rise in chronic diseases, and a higher life expectancy. This scenario poses a monumental challenge for governments, which face the difficult task of addressing unlimited needs with finite resources. This imbalance creates a significant strain on healthcare systems, requiring them to prioritize interventions, reorganize services, and optimize resource management.

Compounding this issue is a critical challenge: the shortage of healthcare professionals, particularly evident in European countries such as Spain. The scarcity of physicians, nurses, and other healthcare workers exacerbates an already difficult situation, increasing the workload on existing staff, extending working hours, and contributing to a highly stressful work environment.

In this context, special attention must be given to the well-being of healthcare professionals, not only because of their central role in patient care but also because their physical, mental, and social health directly influences the quality of care they provide. The stressful conditions of their work—the constant exposure to suffering, limited resources, rotating shifts, and high pressure—heighten their risk of developing conditions such as insulin resistance and other cardiometabolic disorders.

Our study highlights the association between specific risk factors and insulin resistance in this population, underscoring the urgent need to implement preventive measures from a political and administrative perspective. By identifying and addressing these factors, it is possible to not only enhance the health and well-being of healthcare professionals but also optimize the healthcare system as a whole, ultimately benefiting the broader population that relies on it.

### Strengths and Limitations

This study has several strengths, including its large sample size (nearly 45,000 workers), making it one of the largest studies on healthcare workers worldwide. Additionally, it is among the first, if not the very first, to assess the prevalence of high IR risk across different healthcare roles. The wide range of variables analyzed—sociodemographic and lifestyle-related—combined with its longitudinal design, provides a foundation for establishing causal relationships.

A limitation of the study is the exclusion of unemployed individuals, retirees, those under 18, and those over 69 years of age. While this exclusion restricts the generalizability to the broader population, we believe the large sample size mitigates this impact. Another limitation is the absence of confounding factors such as comorbidities or pharmacological treatments, due to the unavailability of such data. Another limitation is that the study population consists exclusively of healthcare workers, who present unique characteristics compared to other types of workers. Consequently, our results may not be generalizable to the broader working population.

## 5. Conclusions

In summary, various sociodemographic and lifestyle factors are associated with the risk of insulin resistance, a condition linked to the development of metabolic and cardiovascular diseases. Age, sex, socioeconomic status, smoking, physical activity, and the adherence to the Mediterranean diet are key determinants that collectively seem to influence insulin sensitivity. The use of tools such as the TyG, SPISE, and METS-IR indices has enabled a precise evaluation of IR, particularly in groups such as healthcare workers, where job demands and lifestyle significantly impact metabolic health. As the prevalence of IR continues to rise, it is crucial to implement comprehensive interventions targeting these risk factors in a personalized manner.

## Figures and Tables

**Table 1:** Characteristics of the population.

	Men n = 14,305	Women n = 30,686	Total n = 44,991	
	**Mean (SD)**	**Mean (SD)**	**Mean (SD)**	***p*-value**
Age (years)	41.1 (10.6)	40.4 (10.5)	40.6 (10.5)	<0.001
Height (cm)	176.0 (7.5)	162.6 (6.0)	166.8 (9.0)	<0.001
Weight (kg)	81.2 (14.5)	63.7 (13.3)	69.3 (15.9)	<0.001
Waist circumference (cm)	89.7 (12.6)	76.7 (11.8)	80.8 (13.5)	<0.001
Hip circumference (cm)	101.7 (8.8)	99.3 (10.7)	100.1 (10.2)	<0.001
Systolic blood pressure (mmHg)	128.2 (13.1)	116.1 (13.8)	119 9 (14.7)	<0.001
Diastolic blood pressure (mmHg)	79.9 (10.6)	74.8 (10.1)	76.4 (10.5)	<0.001
Total cholesterol (mg/dL)	191.8 (37.2)	187.8 (34.6)	189.1 (35.5)	<0.001
HDL-c (mg/dL)	48.9 (11.2)	59.3 (12.8)	56.0 (13.2)	<0.001
LDL-c (mg/dL)	165.2 (46.2)	144.8 (38.9)	151.3 (42.4)	<0.001
Triglycerides (mg/dL)	111.0 (73.2)	81.7 (47.0)	91.0 (58.3)	<0.001
Glucose (mg/dL)	93.6 (18.2)	88.9 (12.4)	90.4 (14.7)	<0.001
AST (U/L)	24.1 (17.2)	18.2 (8.0)	20.1 (12.1)	<0.001
ALT (U/L)	29.0 (36.7)	17.3 (13.7)	21.0 (24.2)	<0.001
GGT (U/L)	30.2 (28.8)	18.1 (18.1)	22.0 (22.7)	<0.001
	**N (%)**	**N (%)**		***p*-value**
<30 years	2400 (16.8)	5984 (19.5)	8384 (18.6)	<0.001
30–39 years	4200 (29.4)	8304 (27.1)	12,504 (27.8)	
40–49 years	4512 (31.5)	10,128 (33.0)	14,640 (32.5)	
50–59 years	2449 (17.1)	5150 (16.8)	7599 (16.9)	
60–69 years	744 (5.2)	1120 (3.6)	1864 (1.1)	
Physicians	5064 (35.4)	5024 (16.4)	10,088 (22.4)	<0.001
Nurses	4008 (28.0)	12,752 (41.6)	16,760 (37.3)	
Health technicians	1728 (12.1)	4128 (13.5)	5856 (13.0)	
Nursing assistants or orderlies	3505 (24.5)	8782 (28.5)	12,287 (27.3)	
Non-smokers	12,001 (83.9)	26,094 (85.0)	38,095 (84.7)	<0.001
Smokers	2304 (16.1)	4592 (15.0)	6896 (15.3)	
No physical activity	7512 (52.5)	18,744 (61.1)	26,256 (58.4)	<0.001
Physical activity	6793 (47.5)	11,942 (38.9)	18,735 (41.6)	
Non-Mediterranean diet	7771 (54.2)	19,243 (62.7)	27,014 (60.0)	<0.001
Mediterranean diet	6534 (45.8)	11,443 (37.3)	17,977 (40.0)	

HDL—high density lipoprotein. LDL—low density lipoprotein. AST—aspartate aminotransferase. ALT—alanine aminotransferase. GGT—gamma-glutamyl transpeptidase. SD—standard deviation.

**Table 2:** Mean values of insulin resistance risk scales according to sociodemographic variables and healthy habits by sex.

		TyG Index		METS-IR		SPISE-IR	
**Men**	**n**	**Mean (SD)**	***p*-Value**	**Mean (SD)**	***p*-Value**	**Mean (SD)**	***p*-Value**
<30 years	2400	8.0 (0.4)	<0.001	34.1 (7.6)	<0.001	1.4 (0.4)	<0.001
30–39 years	4200	8.3 (0.5)		37.3 (7.8)		1.6 (0.5)	
40–49 years	4512	8.4 (0.5)		40.0 (8.3)		1.7 (0.5)	
50–59 years	2449	8.6 (0.6)		42.4 (8.4)		1.8 (0.5)	
60–69 years	744	8.7 (0.6)		43.3 (9.3)		1.9 (0.6)	
Physicians	5064	8.3 (0.5)	<0.001	37.8 (7.5)	<0.001	1.6 (0.4)	<0.001
Nurses	4008	8.3 (0.6)		38.0 (8.7)		1.6 (0.5)	
Health technicians	1728	8.4 (0.5)		38.7 (9.2)		1.7 (0.6)	
Nursing assistants or orderlies	3505	8.5 (0.6)		41.5 (9.7)		1.8 (0.6)	
Non-smokers	12,001	8.3 (0.6)	<0.001	38.6 (8.6)	<0.001	1.6 (0.5)	<0.001
Smokers	2304	8.5 (0.7)		40.1 (9.4)		1.7 (0.6)	
No physical activity	7512	8.5 (0.6)	<0.001	41.3 (9.5)	<0.001	1.8 (0.6)	<0.001
Physical activity	6793	8.2 (0.5)		36.2 (7.0)		1.5 (0.4)	
Non-Mediterranean diet	7771	8.5 (0.6)	<0.001	40.8 (9.3)	<0.001	1.7 (0.6)	<0.001
Mediterranean diet	6534	8.3 (0.6)		36.9 (7.1)		1.5 (0.4)	
**Women**	**n**	**Mean (SD)**	***p*-value**	**Mean (SD)**	***p*-value**	**Mean (SD)**	***p*-value**
<30 years	5984	7.9 (0.4)	<0.001	29.9 (5.7)	<0.001	1.2 (0.3)	<0.001
30–39 years	8304	8.0 (0.4)		31.8 (8.3)		1.3 (0.5)	
40–49 years	10,128	8.1 (0.4)		34.2 (8.0)		1.4 (0.5)	
50–59 years	5150	8.3 (0.5)		36.7 (9.1)		1.5 (0.5)	
60–69 years	1120	8.5 (0.5)		37.0 (10.0)		1.6 (0.6)	
Physicians	5024	7.9 (0.4)	<0.001	29.5 (5.3)	<0.001	1.1 (0.3)	<0.001
Nurses	12,752	8.0 (0.4)		31.8 (7.4)		1.3 (0.4)	
Health technicians	4128	8.2 (0.5)		35.2 (8.8)		1.4 (0.5)	
Nursing assistants or orderlies	8782	8.3 (0.5)		36.5 (9.4)		1.5 (0.6)	
Non-smokers	26,094	8.0 (0.5)	<0.001	33.0 (8.1)	<0.001	1.3 (0.5)	<0.001
Smokers	4592	8.2 (0.5)		34.6 (9.6)		1.4 (0.6)	
No physical activity	18,744	8.2 (0.5)	<0.001	34.5 (9.1)	<0.001	1.4 (0.5)	<0.001
Physical activity	11,942	8.0 (0.4)		31.2 (6.5)		1.2 (0.4)	
Non-Mediterranean diet	19,243	8.2 (0.5)	<0.001	33.9 (9.4)	<0.001	1.4 (0.5)	<0.001
Mediterranean diet	11,443	8.0 (0.4)		31.9 (6.7)		1.2 (0.5)	
**Total**	**n**	**Mean (SD)**	***p*-value**	**Mean (SD)**	***p*-value**	**Mean (SD)**	***p*-value**
<30 years	8384	8.0 (0.4)	<0.001	31.1 (6.6)	<0.001	1.2 (0.4)	<0.001
30–39 years	12,504	8.1 (0.5)		33.7 (8.5)		1.4 (0.5)	
40–49 years	14,640	8.2 (0.5)		36.0 (8.5)		1.5 (0.5)	
50–59 years	7599	8.4 (0.6)		38.8 (9.7)		1.6 (0.6)	
60–69 years	1864	8.6 (0.6)		39.2 (9.7)		1.7 (0.6)	
Physicians	10,088	8.1 (0.5)	<0.001	33.3 (8.1)	<0.001	1.3 (0.4)	<0.001
Nurses	16,760	8.1 (0.5)		33.7 (7.7)		1.4 (0.5)	
Health technicians	5856	8.2 (0.5)		36.2 (9.0)		1.5 (0.5)	
Nursing assistants or orderlies	12,287	8.3 (0.6)		37.9 (9.8)		1.6 (0.6)	
Non-smokers	38,095	8.1 (0.5)	<0.001	34.8 (8.7)	<0.001	1.4 (0.5)	<0.001
Smokers	6896	8.3 (0.6)		36.4 (9.9)		1.5 (0.6)	
No physical activity	26,256	8.3 (0.5)	<0.001	36.5 (9.7)	<0.001	1.5 (0.6)	<0.001
Physical activity	18,735	8.1 (0.5)		33.0 (7.1)		1.3 (0.4)	
Non-Mediterranean diet	27,014	8.4 (0.5)	<0.001	36.3 (9.6)	<0.001	1.5 (0.5)	<0.001
Mediterranean diet	17,977	8.1 (0.4)		33.2 (7.0)		1.3 (0.5)	

TyG—Triglyceride–Glucose index. METS-IR—Metabolic Score for Insulin Resistance. SPISE-IR—Single-Point Insulin Sensitivity Estimator. SD—standard deviation.

**Table 3:** Prevalence of high values of insulin resistance risk scales according to sociodemographic variables and healthy habits by sex.

		TyG Index High		METS-IR High		SPISE-IR High	
**Men**	**n**	**%**	***p*-Value**	**%**	***p*-Value**	**%**	***p*-Value**
<30 years	2400	4.0	<0.001	3.8	<0.001	4.1	<0.001
30–39 years	4200	16.0		8.1		9.1	
40–49 years	4512	22.9		12.6		17.0	
50–59 years	2449	27.4		22.5		24.5	
60–69 years	744	38.7		22.6		25.8	
Physicians	5064	16.6	<0.001	7.9	<0.001	9.5	<0.001
Nurses	4008	18.6		8.3		12.6	
Health technicians	1728	19.4		10.9		13.9	
Nursing assistants or orderlies	3505	23.3		20.5		23.3	
Non-smokers	12,001	17.6	<0.001	11.1	<0.001	13.6	<0.001
Smokers	2304	27.1		15.6		17.7	
No physical activity	7512	24.0	<0.001	17.8	<0.001	20.4	<0.001
Physical activity	6793	13.8		5.3		7.4	
Non-Mediterranean diet	7771	22.7	<0.001	16.5	<0.001	18.8	<0.001
Mediterranean diet	6534	15.0		7.3		8.9	
**Women**	**n**	**%**	***p*-value**	**%**	***p*-value**	**%**	***p*-value**
<30 years	5984	3.2	<0.001	2.8	<0.001	1.4	<0.001
30–39 years	8304	4.2		3.9		4.8	
40–49 years	10,128	5.1		4.6		6.0	
50–59 years	5150	16.5		9.6		11.2	
60–69 years	1120	22.9		14.3		15.7	
Physicians	5024	2.5	<0.001	2.6	<0.001	1.9	<0.001
Nurses	12,752	3.9		3.1		3.5	
Health technicians	4128	11.6		7.1		9.3	
Nursing assistants or orderlies	8782	12.0		9.1		11.1	
Non-smokers	26,094	6.4	<0.001	4.7	<0.001	5.5	<0.001
Smokers	4592	10.8		5.9		8.7	
No physical activity	18,744	8.2	<0.001	6.6	<0.001	8.1	<0.001
Physical activity	11,942	5.2		2.1		2.7	
Non-Mediterranean diet	19,213	7.8	<0.001	6.0	<0.001	7.5	<0.001
Mediterranean diet	11,413	5.9		2.7		3.4	
**Total**	**n**	**%**	***p*-value**	**%**	***p*-value**	**%**	***p*-value**
<30 years	8384	3.1	<0.001	1.4	<0.001	2.1	<0.001
30–39 years	12,504	8.2		5.2		6.3	
40–49 years	14,640	10.5		7.0		9.4	
50–59 years	7599	20.0		13.8		15.5	
60–69 years	1864	29.2		17.6		19.7	
Physicians	10,088	7.4	<0.001	4.3	<0.001	5.1	<0.001
Nurses	16,760	9.6		5.0		5.7	
Health technicians	5856	13.9		6.8		10.7	
Nursing assistants or orderlies	12,287	15.2		12.4		14.6	
Non-smokers	38,095	9.9	<0.001	6.7	<0.001	8.1	<0.001
Smokers	6896	16.2		9.2		11.7	
No physical activity	26,256	12.7	<0.001	9.8	<0.001	11.6	<0.001
Physical activity	18,735	8.3		3.3		4.4	
Non-Mediterranean diet	27,014	11.8	<0.001	9.4	<0.001	10.9	<0.001
Mediterranean diet	17,977	8.9		3.9		5.1	

TyG—Triglyceride–Glucose index. METS-IR—Metabolic Score for Insulin Resistance. SPISE-IR—Single-Point Insulin Sensitivity Estimator.

**Table 4:** Binary logistic regression.

	TyG Index High	*p*-Value	METS-IR High	*p*-Value	SPISE-IR High	*p*-Value
	OR (95% CI)		OR (95% CI)		OR (95% CI)	
Women	1		1		1	
Men	3.74 (3.51–3.98)	<0.001	3.68 (3.40–3.97)	<0.001	3.59 (3.34–3.84)	<0.001
<30 years	1		1		1	
30–39 years	1.84 (1.63–2.05)	<0.001	1.60 (1.38–1.83)	<0.001	1.65 (1.43–1.87)	<0.001
40–49 years	3.92 (3.47–4.37)	<0.001	3.34 (2.89–3.80)	<0.001	2.82 (2.46–3.19)	<0.001
50–59 years	5.32 (4.68–5.97)	<0.001	4.49 (3.84–5.15)	<0.001	4.30 (3.71–4.90)	<0.001
60–69 years	11.73 (9.95–13.52)	<0.001	12.26 (9.79–14.74)	<0.001	9.54 (7.83–11.25)	<0.001
Physicians	1		1		1	
Nurses	1.11 (1.08–1.14)	<0.001	1.61 (1.46–1.76)	<0.001	1.31 (1.18–1.44)	<0.001
Health technicians	1.32 (1.21–1.43)	<0.001	1.80 (1.60–2.00)	<0.001	1.76 (1.61–1.92)	<0.001
Nursing assistants or orderlies	1.99 (1.82–2.16)	<0.001	4.16 (3.68–4.63)	<0.001	4.08 (3.65–4.51)	<0.001
Non-smokers	1		1		1	
Smokers	1.52 (1.41–1.63)	<0.001	1.19 (1.14–1.24)	<0.001	1.22 (1.15–1.30)	<0.001
Physical activity	1		1		1	
No physical activity	1.64 (1.53–1.74)	<0.001	3.54 (3.23–3.85)	<0.001	3.12 (2.87–3.38)	<0.001
Mediterranean diet	1		1		1	
Non-Mediterranean diet	1.48 (1.39–1.58)	<0.001	2.60 (2.29–2.90)	<0.001	2.30 (2.02–2.58)	<0.001

TyG—Triglyceride–Glucose index. METS-IR—Metabolic Score for Insulin Resistance. SPISE-IR—Single-Point Insulin Sensitivity Estimator. OR—odds ratio.

**Table 5:** Pearson’s correlation coefficients and Cohen’s kappa values of the insulin resistance risk scales.

*Pearson*	TyG Index	SPISE-IR	METS-IR
TyG index	1	0.681	0.621
SPISE-IR		1	0.986
METS-IR			1
***kappa Cohen***	**TyG index high**	**SPISE-IR high**	**METS-IR high**
TyG index high	1	0.485	0.402
SPISE-IR high		1	0.849
METS-IR high			1

TyG—Triglyceride–Glucose index. METS-IR—Metabolic Score for Insulin Resistance. SPISE-IR—Single-Point Insulin Sensitivity Estimator.

**Table 6:** Differences in the prevalences of high values of insulin resistance risk scales between the pre- and post-study period by sex.

		TyG Index High			SPISE-IR High			METS-IR High		
**Men**	**n**	**%Pre-Post**	**Difference %**	***p*-Value**	**%Pre-Post**	**Difference %**	***p*-Value**	**%Pre-Post**	**Difference %**	***p*-Value**
<30 years	2400	3.8–4.0	4.1	<0.001	4.2–4.4	4.4	<0.001	3.9–4.1	4.6	<0.001
30–39 years	4200	14.7–16.0	7.9		7.5–8.2	8.2		8.3–9.1	8.6	
40–49 years	4512	20.1–22.9	12.3		12.2–13.5	9.3		15.5–17.0	11.9	
50–59 years	2449	23.3–27.4	14.9		13.8–15.6	11.8		20.8–24.5	15.2	
60–69 years	744	31.4–38.7	18.9		16.5–19.9	17.3		20.6–25.8	20.2	
Physicians	5064	15.8–16.6	4.7	<0.001	5.3–5.5	4.1	<0.001	8.9–9.5	5.8	<0.001
Nurses	4008	17.5–18.6	5.9		6.5–6.8	4.7		11.7–12.6	7.5	
Health technicians	1728	17.4–19.4	10.3		9.9–10.8	8.2		12.1–13.9	12.9	
Nursing assistants or orderlies	3505	19.4–23.3	16.9		16.7–18.9	11.5		18.9–23.3	18.8	
Non-smokers	12,001	16.3–17.6	7.2	<0.001	8.9–9.6	7.0	<0.001	12.4–13.6	8.9	<0.001
Smokers	2304	23.6–27.1	12.8		11.6–12.8	9.1		15.6–17.7	11.6	
No physical activity	7512	19.8–24.0	17.3	<0.001	15.6–18.6	16.3	<0.001	16.3–20.4	19.9	<0.001
Physical activity	6793	13.3–13.8	3.8		4.1–4.3	4.4		7.0–7.4	5.6	
Non-Mediterranean diet	7771	19.0–22.7	16.5	<0.001	14.3–16.9	15.1	<0.001	15.4–18.8	18.1	<0.001
Mediterranean diet	6534	14.3–15.0	4.6		5.2–5.5	5.1		8.3–8.9	6.2	
**Women**	**n**	**%pre-post**	**difference %**	***p*-value**	**%pre-post**	**difference %**	***p*-value**	**%pre-post**	**difference %**	***p*-value**
<30 years	5984	3.1–3.2	2.9	<0.001	2.7–2.8	3.3	<0.001	1.3–1.4	4.1	<0.001
30–39 years	8304	4.0–4.2	5.5		3.7–3.9	6.1		4.5–4.8	6.9	
40–49 years	10,128	4.6–5.1	9.1		4.1–4.6	10.5		5.3–6.0	11.5	
50–59 years	5150	14.7–16.5	11.2		8.3–9.6	13.9		9.5–11.2	14.8	
60–69 years	1120	19.6–22.9	14.6		11.8–14.3	17.3		12.9–15.7	17.9	
Physicians	5024	2.4–2.5	3.6	<0.001	2.5–2.6	5.2	<0.001	1.8–1.9	6.3	<0.001
Nurses	12,752	3.7–3.9	4.9		2.9–3.1	6.8		3.2–3.5	7.9	
Health technicians	4128	10.6–11.6	8.9		6.4–7.1	10.2		8.3–9.3	10.8	
Nursing assistants or orderlies	8782	10.4–12.0	13.3		7.8–9.1	13.8		9.4–11.1	14.9	
Non-smokers	26,094	6.1–6.4	5.9	<0.001	4.3–4.7	7.9	<0.001	5.0–5.5	8.8	<0.001
Smokers	4592	9.8–10.8	9.1		5.2–5.9	11.3		7.7–8.7	11.2	
No physical activity	18,744	6.8–8.2	16.5	<0.001	5.4–6.6	17.8	<0.001	6.6–8.1	18.2	<0.001
Physical activity	11,942	4.9–5.2	5.3		2.0–2.1	5.9		2.5–2.7	6.3	
Non-Mediterranean diet	19,213	6.6–7.8	15.9	<0.001	5.0–6.0	16.8	<0.001	6.5–7.5	17.5	<0.001
Mediterranean diet	11,413	5.5–5.9	6.3		2.5–2.7	6.6		3.1–3.4	7.4	
**Total**	**n**	**%pre-post**	**difference %**	***p*-value**	**%pre-post**	**difference %**	***p*-value**	**%pre-post**	**difference %**	***p*-value**
<30 years	8384	3.4–3.5	3.4	<0.001	3.4–3.5	3.6	<0.001	2.8–2.9	3.9	<0.001
30–39 years	12,504	7.6–8.1	6.2		5.4–5.8	6.4		4.8–5.2	6.7	
40–49 years	14,640	9.4–10.6	10.8		8.0–8.9	10.3		7.9–8.9	10.9	
50–59 years	7599	16.4–18.9	12.9		10.2–11.8	13.3		11.0–12.9	14.1	
60–69 years	1864	23.9–28.6	16.2		14.7–17.8	17.1		15.3–18.7	17.9	
Physicians	10,088	9.1–9.5	4.0	<0.001	3.9–4.1	4.2	<0.001	5.5–5.8	4.5	<0.001
Nurses	16,760	9.6–10.2	5.2		4.7–5.0	5.5		6.5–6.9	5.8	
Health technicians	5856	12.6–14.0	9.4		8.0–8.9	9.9		9.6–10.8	10.9	
Nursing assistants or orderlies	12,287	5.5–6.5	14.2		12.2–14.4	15.0		12.7–15.3	16.7	
Non-smokers	38,095	8.7–9.3	6.3	<0.001	6.6–7.1	6.5	<0.001	6.6–7.1	6.7	<0.001
Smokers	6896	13.9–15.6	10.5		9.0–10.1	10.9		9.6–10.8	11.0	
No physical activity	26,256	12.4–14.9	16.8	<0.001	9.7–11.8	17.6	<0.001	10.0–12.2	18.1	<0.001
Physical activity	18,735	6.7–7.1	4.8		3.7–3.9	5.1		6.4–6.8	5.5	
Non-Mediterranean diet	27,014	11.7–14.0	16.2	<0.001	9.1–11.0	16.9	<0.001	9.7–11.8	17.3	<0.001
Mediterranean diet	17,977	7.3–7.7	5.4		4.8–5.1	5.8		6.7–7.2	6.2	

TyG—Triglyceride–Glucose index. METS-IR—Metabolic Score for Insulin Resistance. SPISE-IR—Single-Point Insulin Sensitivity Estimator. Pre-year 2010. Post-year 2019.

## Data Availability

The data are not available due to ethical or privacy restrictions.
